# Integration of Clinical, Cytokine, and Ultrasound Data Using Machine Learning Reveals a Multidimensional Inflammatory Signature in Polymyalgia Rheumatica

**DOI:** 10.3390/jpm16080414

**Published:** 2026-08-02

**Authors:** Christian D’Elia, Edda Russo, Giada Santagata, Riccardo Terenzi, Francesca Li Gobbi, Emanuele Antonio Maria Cassarà, Elisa Cioffi, Valentina Grossi, Francesca Romano, Barbara Lari, Maria Infantino, Mariangela Manfredi, Serena Guiducci, Maurizio Benucci

**Affiliations:** 1 Department of Experimental and Clinical Medicine, Division of Rheumatology, University of Florence, 50134 Florence, Italy; christian.delia@unifi.it (C.D.); giada.santagata@unifi.it (G.S.); serena.guiducci@unifi.it (S.G.); 2 Clinical Pathology Laboratory Unit, S. Giuseppe Hospital, Azienda USL Toscana Centro, 50053 Empoli, Italy; 3 Rheumatology Unit, S. Giovanni di Dio Hospital, Azienda USL-Toscana Centro, 50143 Florence, Italy; riccardo.terenzi@uslcentro.toscana.it (R.T.); francesca.ligobbi@uslcentro.toscana.it (F.L.G.); emanueleantonio.cassara@uslcentro.toscana.it (E.A.M.C.); elisa.cioffi@uslcentro.toscana.it (E.C.); maurizio.benucci@uslcentro.toscana.it (M.B.); 4 Immunology and Allergology Laboratory Unit, S. Giovanni di Dio Hospital, Azienda USL-Toscana Centro, 50143 Florence, Italy; valentina2.grossi@uslcentro.toscana.it (V.G.); barbara.lari@uslcentro.toscana.it (B.L.); maria2.infantino@uslcentro.toscana.it (M.I.); mariangela.manfredi@uslcentro.toscana.it (M.M.); 5 General Laboratory, Careggi University Hospital, 50134 Florence, Italy; romanofr@aou-careggi.toscana.it

**Keywords:** polymyalgia rheumatica, artificial intelligence, machine learning, cytokines, serum amyloid A, ultrasound, disease activity, personalized medicine

## Abstract

**Background**: Polymyalgia rheumatica (PMR) is a clinically heterogeneous inflammatory disorder in which conventional acute-phase reactants may inadequately reflect the complexity and biological variability of disease activity. Precision medicine approaches integrating multidimensional clinical and laboratory data may offer a more comprehensive characterization of inflammatory phenotypes. This study investigated whether the combined assessment of cytokine profiles, routine laboratory biomarkers, ultrasound findings, and clinical variables could improve disease activity stratification in PMR. **Methods**: A total of 103 consecutive patients with PMR were retrospectively analyzed. Disease activity was assessed using the PMR Activity Score (PMR-AS) and, for exploratory purposes, dichotomized according to the cohort median (≥11 vs. <11). Group differences were evaluated using non-parametric statistical methods. The multidimensional structure of the dataset was explored through Spearman correlation analysis, principal component analysis (PCA), and unsupervised clustering. Predictive models, including logistic regression, random forest, and gradient boosting, were developed to evaluate the potential contribution of integrated analytical approaches to patient stratification. **Results**: The study cohort included 103 patients (63.1% female), with a median age of 76 years (IQR 71–81); 57 patients (55.3%) presented PMR-AS ≥11. Patients with higher disease activity exhibited significantly increased levels of CRP, fibrinogen, platelet count, IL-6, serum amyloid A (SAA), and myeloid-related protein (MRP), together with a higher prevalence of joint effusion and Power Doppler positivity. Among the evaluated biomarkers, SAA demonstrated the strongest correlation with disease activity (Spearman ρ = 0.878; *p* < 0.001). Multivariate predictive modeling showed high discriminative performance, with random forest achieving the highest cross-validated AUC (0.934), whereas gradient boosting demonstrated the best overall accuracy (0.883). Unsupervised clustering analysis identified a subgroup characterized by a more pronounced inflammatory signature associated with higher PMR-AS values. **Conclusions**: These findings support the concept that disease activity in PMR may be more effectively represented through an integrated multidimensional inflammatory profile rather than isolated biomarkers. The combined evaluation of routine laboratory parameters, cytokines, and imaging features may contribute to more refined patient stratification within a precision medicine framework. Although exploratory, these results highlight the potential value of advanced data integration strategies for supporting biologically informed disease characterization in PMR, while underscoring the need for external validation before translation into clinical practice.

## 1. Introduction

Polymyalgia rheumatica (PMR) is a common inflammatory disorder occurring in older adults [1], characterized by proximal pain and stiffness, elevated inflammatory markers, and a rapid response to glucocorticoids. Despite its well-recognized clinical presentation, the assessment of disease activity remains largely based on composite clinical scores and conventional laboratory parameters, such as erythrocyte sedimentation rate (ESR) and C-reactive protein (CRP) [2].

While these markers are widely used in clinical practice, they provide only a partial representation of the underlying inflammatory processes and may not adequately capture disease heterogeneity. Increasing evidence indicates that PMR is driven by a complex interplay of innate immune activation and cytokine signaling, with interleukin-6 (IL-6) playing a central role in disease pathophysiology [3].

Elevated IL-6 levels have been consistently associated with disease activity and therapeutic response, and the clinical efficacy of IL-6-targeted therapies further supports this role [4,5].

However, beyond IL-6, the contribution of additional inflammatory mediators—including acute-phase proteins such as serum amyloid A (SAA)—remains insufficiently characterized in PMR, despite evidence supporting its role as a sensitive marker of systemic inflammation [6].

An additional element of complexity in PMR is represented by the diagnostic challenge. The absence of specific clinical signs, unique biomarkers, or definitive instrumental tests often makes the diagnosis reliant on clinical criteria and the exclusion of mimicking conditions, including inflammatory rheumatic diseases, infections, and malignancies [7].

The assessment of disease activity also represents a significant challenge. Although the PMR Activity Score (PMR-AS) is widely used as a composite measure of disease activity, its performance, while good, is not without limitations, and there is still no definitive consensus on the optimal cut-off values to distinguish active disease from remission. Recent prospective studies have demonstrated the high discriminative ability of the PMR-AS, but with variability in cut-off values depending on disease stage and clinical context, suggesting that even composite scores may not fully capture the heterogeneity of PMR [5,8].

Moreover, the reliance on parameters such as C-reactive protein (CRP) represents an additional limitation, particularly in patients treated with therapies that directly modulate the systemic inflammatory response, in whom CRP levels may not accurately reflect clinical disease activity [9,10].

In this context, imaging modalities have assumed an increasingly important role not only in diagnosis but also in disease characterization, allowing the assessment of articular, periarticular, and extra-articular involvement [11].

Recent studies have demonstrated a significant prevalence of involvement of extra-articular structures, such as bursae, tendons, and entheses, suggesting that PMR is primarily a disease of extra-articular tissues [12].

Recent evidence suggests that, beyond alterations in circulating cytokines, PMR is characterized by a compartmentalized immune response, in which T-cell subsets show a distinct distribution between peripheral blood and inflamed tissues. In particular, while TH17 cells appear increased in the peripheral circulation, a predominance of a TH1 signature has been identified in synovial fluid and bursal tissue, supporting a role for IFN-γ–mediated inflammation at the site of disease. This suggests that circulating biomarkers may only partially reflect the inflammatory processes occurring at the disease site [13].

In parallel, the integration of imaging findings, particularly ultrasound-based assessment of synovial and periarticular inflammation, has improved diagnostic accuracy and disease characterization [14,15,16].

Ultrasound, in particular, was included in the 2012 EULAR/ACR classification criteria, representing the first attempt to integrate imaging data into the diagnostic process of PMR. Findings such as bilateral subacromial-subdeltoid bursitis and tenosynovitis of the long head of the biceps are considered typical, although not specific [11].

However, the simple binary assessment of the presence or absence of synovitis, bursitis, or tenosynovitis may not be sufficient to distinguish PMR from mimicking conditions, particularly elderly-onset rheumatoid arthritis with polymyalgic presentation. In this context, more refined ultrasound approaches, based on a semiquantitative assessment of the Power Doppler signal and its anatomical distribution, may provide additional information. Suzuki et al. have indeed observed that, in patients with PMR, inflammation tends to localize predominantly in extrasynovial soft tissues adjacent to the subscapularis tendon, rather than in the joint synovium itself. Although these findings require further confirmation, they suggest that ultrasound imaging may contribute not only to differential diagnosis but also to the topographic characterization of the inflammatory process. In this perspective, ultrasound may provide quantitative and spatial information useful for a more precise definition of the inflammatory phenotype of PMR [17].

Nevertheless, these modalities are still not routinely incorporated into integrated models of disease activity, and their relationship with circulating inflammatory mediators remains incompletely defined. Taken together, these observations highlight a key limitation of current approaches: PMR activity is typically evaluated using a limited set of variables, often considered in isolation, rather than within an integrated biological framework. This reductionist approach may obscure relevant interactions between systemic inflammation, cytokine networks, and imaging features, ultimately limiting the ability to stratify patients according to their true inflammatory burden.

In recent years, artificial intelligence (AI) and machine learning techniques have emerged as useful tools for the analysis of complex, high-dimensional biomedical data. In rheumatology, these approaches have been increasingly explored to identify disease patterns, support clinical decision-making, and move toward more individualized strategies [18,19]. However, their application in PMR remains limited, particularly in the context of integrating clinical, laboratory, cytokine, and imaging data within a unified analytical framework.

The present study was designed to address this gap by applying machine learning methods to a well-characterized cohort of patients with PMR. By integrating conventional inflammatory markers, cytokine profiles, and ultrasound findings, we aimed to (i) identify the variables most strongly associated with disease activity, (ii) evaluate the ability of data-driven models to discriminate between different levels of clinical activity, and (iii) explore the presence of underlying inflammatory patterns within the dataset.

We hypothesized that PMR activity reflects a complex inflammatory network rather than the isolated behavior of single biomarkers. Accordingly, integrated analytical models combining laboratory, cytokine, imaging, and clinical variables may better capture disease heterogeneity and support a more personalized approach to patient stratification.

## 2. Materials and Methods

### 2.1. Study Design and Dataset

This retrospective observational study included 103 patients with PMR enrolled at a single center (Rheumatology Department, San Giovanni di Dio Hospital, Florence, Italy) between 2025- September and 2026 April. The dataset comprised demographic characteristics, conventional inflammatory biomarkers, cytokine measurements, ultrasound variables, and disease activity data collected during routine clinical assessment. All patients were evaluated within 4 weeks of symptom onset in the outpatient clinic (fast track) of the Rheumatology Unit at S. Giovanni di Dio Hospital in Florence. At the time of the visit, patients were not being treated with corticosteroids, DMARDs, or b-DMARDs. The working dataset contained no missing values. For the purposes of this manuscript, the data were analyzed in de-identified form and used to develop an exploratory proof-of-concept framework for personalized disease stratification.

### 2.2. Variables and Outcome Definition

The following variables were available for analysis: sex, age, symptom duration (weeks), erythrocyte sedimentation rate (ESR), C-reactive protein (CRP), fibrinogen, platelet count, interleukin-6 (IL-6), serum amyloid A (SAA), myeloid-related protein (MRP), IL-17, IL-1β, joint effusion, synovial hypertrophy, Power Doppler signal, and polymyalgia rheumatica activity score (PMR-AS). All blood samples used for laboratory biomarker assessment were collected at the time of the baseline clinical and ultrasound evaluation, within four weeks of symptom onset and before the initiation of corticosteroids, conventional synthetic DMARDs, or biologic DMARDs. ESR was measured using an automated system (Alifax, Padova, Italy), and CRP levels were determined by immunoturbidimetric assay on a UniCel DxC 800 Synchron system (Beckman Coulter Inc., Brea, CA, USA). Fibrinogen levels and platelet count were assessed using routine automated coagulation and hematology analyzers, respectively, according to standard laboratory procedures. Serum cytokines, including IL-6 ((Beckman Coulter Inc., Brea, CA, USA), IL-17 (R&D Systems, Minneapolis, MN, USA), and IL-1β (eBioscience/Bender MedSystem GmbH, Vienna, Austria), were measured using commercially available enzyme-linked immunosorbent assay (ELISA) kits. The lower limits of detection of the ELISAs were 0.1 pg/mL for both IL-1β and IL-17. Serum amyloid A (SAA) was measured using an immunonephelometric method on a Nephelometer BNII (Siemens Healthcare, Erlangen, Germany) and MRP levels were determined using ELISA immunoassay on Impatto twin Plus BRC (Eurospital, Trieste, Italy). The primary outcome was high disease activity, defined as PMR-AS ≥ 11. This threshold corresponded to the sample median and was selected to obtain a balanced binary outcome for exploratory classification modeling. Accordingly, this dichotomization should be interpreted as data-driven and hypothesis-generating rather than as an externally validated clinical cutoff.

To provide complementary perspectives on disease activity, conventional statistical analyses and machine learning methods were jointly applied, allowing both hypothesis-driven and data-driven evaluation of the dataset.

### 2.3. Ultrasound Assessment

Ultrasound examinations were performed as part of the routine clinical assessment in all patients. The assessment included the evaluation of joint effusion, synovial hypertrophy, and Power Doppler signal. Ultrasound evaluation was performed bilaterally at the shoulders and the long head of the biceps tendon. All examinations were performed by a single experienced operator using a US was performed using an Esaote (Genoa, Italy) MyLab Twice machine equipped with linear 4–13 and 6–18 MHz. Ultrasound findings were recorded according to (EULAR/OMERACT definitions). US findings were recorded as follows: gleno-humeral (GH) effusion (JE)/synovial hypertrophy (SH), subacro mion-subdeltoid (SA-SD) bursitis, long head of biceps tendon tenosynovitis (LHBT), wrist JE/SH, power Doppler (PD) signal on shoulder (any site) and wrist, metacarpophalangeal JE/SH, hip JE/SH, trochanteric bursitis. Given the retrospective nature of the study, the US data were collected with a simplified method; gray-scale US data were collected as a whole for JE and SH, grading the articular involvement in a three-point scale (0 = absence of abnormalities, 1 = monolateral JE or SH, 2 = bilateral JE or SH), whereas information on PD signal was collected only for shoulder and wrist, with a three-point scale (0 = absence of PD signal, 1 = monolateral PD signals, 2 = bilateral PD signals).

### 2.4. Statistical Analysis

Continuous variables were assessed for distributional characteristics using the Shapiro–Wilk test. As most biomarkers showed non-normal distributions, continuous data are reported as median and interquartile range (IQR), whereas categorical variables are presented as counts and percentages. Comparisons between patients with PMR-AS < 11 and those with PMR-AS ≥ 11 were performed using the Mann–Whitney U test for continuous variables and Fisher’s exact test for categorical variables, as appropriate. Associations between individual variables and PMR-AS were evaluated using Spearman rank correlation coefficients. All statistical tests were two-sided, and *p*-values < 0.05 were considered statistically significant.

### 2.5. Machine-Learning Workflow

Supervised machine-learning models were developed to classify patients according to disease activity, defined for exploratory purposes as PMR-AS ≥ 11. Three classification algorithms were evaluated: logistic regression, random forest, and gradient boosting.

Before model development, the analytical dataset was examined for missing values and variable consistency. No missing values were identified; therefore, no imputation procedure was required. Patient identifiers were excluded from all analyses. Sex was binary encoded as female versus male, whereas all remaining predictors were treated as numeric variables. No a priori feature selection or dimensionality reduction was performed before supervised model development. All available demographic, clinical, laboratory, cytokine, and ultrasound variables were included to preserve the exploratory nature of the analysis and allow the models to evaluate their complementary predictive contributions.

Because logistic regression is sensitive to differences in predictor scale, continuous variables were standardized using z-score transformation. Standardization was incorporated within a scikit-learn pipeline and was fitted separately within each training fold, thereby preventing information leakage from the validation observations. The binary sex variable was not standardized. No scaling was applied to random forest or gradient boosting because tree-based algorithms are not materially affected by differences in predictor scale.

Hyperparameters were predefined a priori, and no automated hyperparameter optimization was performed. This strategy was selected because of the moderate sample size and exploratory study design, to limit model-selection optimism and reduce the risk of overfitting. Logistic regression was implemented with L2 regularization, a regularization parameter of C = 1.0, the limited-memory Broyden–Fletcher–Goldfarb–Shanno solver, and a maximum of 1000 iterations. The random forest model included 100 classification trees, the Gini impurity criterion, bootstrap sampling, unrestricted tree depth, a minimum of two observations required to split an internal node, a minimum of one observation per terminal node, and square-root feature sampling at each split. Gradient boosting was implemented using 100 boosting stages, a learning rate of 0.1, a maximum individual-tree depth of 3, a minimum of two observations required to split an internal node, a minimum of one observation per terminal node, and a subsampling fraction of 1.0. A fixed random seed of 42 was used for stochastic procedures to support computational reproducibility.

Model performance was evaluated using stratified five-fold cross-validation with random shuffling. Stratification preserved the distribution of patients with PMR-AS ≥ 11 and PMR-AS < 11 across the validation folds. During each iteration, four folds were used for model training and the remaining fold for validation, so that every patient contributed once to the out-of-fold validation set.

Out-of-fold predicted probabilities were aggregated across the five validation folds and used to construct receiver operating characteristic curves and calculate the area under the receiver operating characteristic curve. Binary class predictions were obtained using a probability threshold of 0.50. Accuracy, sensitivity, specificity, and F1-score were calculated from the aggregated out-of-fold predictions rather than from predictions generated on the complete training dataset.

For model interpretation, the random forest algorithm was refitted on the complete dataset only after completion of cross-validation. Impurity-based feature-importance estimates were then calculated to describe the relative contribution of the predictors within the fitted model. These estimates were interpreted exclusively as measures of predictive usefulness within the specific analytical framework and not as evidence of biological hierarchy, superiority, or causal relevance.

Given the exploratory design, moderate sample size, and absence of an independent external validation cohort, the machine-learning findings should be regarded as internally validated and hypothesis-generating. External validation will be required before the models or individual predictor rankings can be translated into clinical practice.

As a sensitivity analysis, the machine-learning workflow was repeated using PMR-AS as a continuous outcome. A random forest regression model was developed using the same predictor set and cross-validation strategy. The ranking of predictor importance obtained from the regression model was qualitatively compared with that derived from the primary classification analysis to evaluate the robustness of the identified inflammatory signature.

### 2.6. Exploratory Multivariate Analyses

To investigate the latent structure of the dataset, principal component analysis (PCA) was performed on standardized numeric variables using z-score scaling. PCA was used as an unsupervised dimensionality-reduction approach to examine covariance patterns across inflammatory, cytokine, and clinical variables. In addition, unsupervised k-means clustering (k = 2; random_state = 42) was applied to inflammatory, cytokine, and ultrasound variables to explore the presence of major inflammatory phenotypes within the cohort. Cluster assignments were subsequently interpreted in relation to PMR-AS distribution and to the broader biomarker profile of each subgroup. These analyses were exploratory and were intended to support biological pattern recognition rather than formal phenotype validation.

### 2.7. Software

All analyses were performed in Python (version 3.13) in a reproducible computational environment. Data preprocessing and data management were carried out with pandas (version 2.x), statistical analyses with SciPy (version 1.x), machine-learning workflows with scikit-learn (version 1.x), and graphical outputs with matplotlib (version 3.x). Fixed random seeds were used where applicable to improve computational reproducibility. All analytical outputs, summary statistics, and graphical results were reviewed by the investigators for internal consistency with the source dataset.

## 3. Results

### 3.1. Patient Characteristics and Stratification by Disease Activity

The study included 103 patients with PMR. Based on the predefined data-driven cutoff, 57 patients (55.3%) were classified as having high disease activity (PMR-AS ≥ 11) and 46 patients (44.7%) as having lower disease activity (PMR-AS < 11). In the overall cohort, the median PMR-AS was 11 (interquartile range [IQR], 6–14). Baseline comparisons between the two groups are summarized in Table 1. No significant differences were observed for age or sex distribution, indicating that the two activity groups were not primarily separated by demographic factors. Likewise, synovial hypertrophy, IL-17, and IL-1β did not significantly differ between groups. In contrast, patients with PMR-AS ≥ 11 showed a markedly more inflammatory profile. The strongest between-group difference was observed for SAA, which was substantially higher in the high-activity group (median 77.0, IQR 45.5–192.0) than in the low-activity group (median 10.0, IQR 6.9–20.9; *p* < 0.001). Similarly, CRP was significantly higher in patients with high disease activity (median 1.91, IQR 0.94–3.48) compared with those with lower activity (median 0.36, IQR 0.18–0.92; *p* < 0.001). IL-6 also differed significantly between groups (median 9.8, IQR 6.6–21.8 vs. 3.75, IQR 3.4–5.8; *p* < 0.001). Additional significant differences were observed for fibrinogen, platelet count, ESR, and MRP, all of which were higher in the high-activity subgroup. Among imaging variables, Power Doppler and joint effusion also showed significant associations with higher PMR-AS, whereas synovial hypertrophy did not.

Overall, these findings support the presence of a biologically coherent high-inflammatory phenotype in patients with elevated clinical activity.

### 3.2. Correlation Analysis

**Figure 1:**
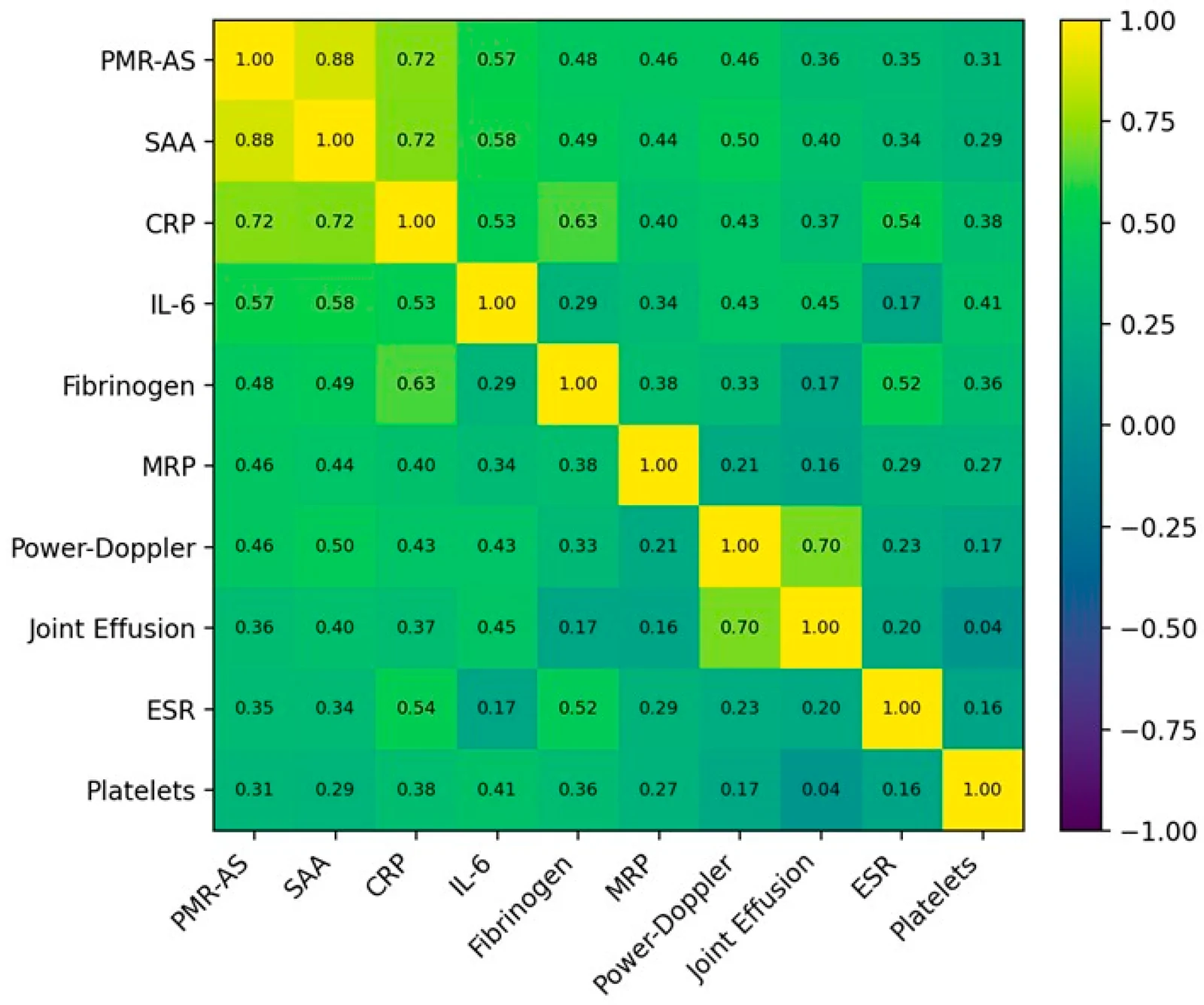
Spearman correlation heatmap among PMR-AS and key inflammatory variables. Darker positive values indicate stronger direct associations. SAA shows the strongest correlation with PMR-AS, followed by CRP, IL-6, fibrinogen, MRP, and Power-Doppler signal.

To further define the relationship between individual variables and disease activity, correlations with PMR-AS were assessed using Spearman rank coefficients. As shown in Figure 1, the correlation structure revealed a coherent inflammatory network linking acute-phase reactants, cytokines, and imaging findings. Among all variables, SAA showed the strongest positive correlation with PMR-AS (rho = 0.878, *p* < 0.001), indicating a very strong monotonic relationship with disease activity. CRP was the second most strongly correlated variable (rho = 0.722, *p* < 0.001), followed by IL-6 (rho = 0.568, *p* < 0.001), fibrinogen (rho = 0.476, *p* < 0.001), MRP (rho = 0.461, *p* < 0.001), and Power Doppler signal (rho = 0.461, *p* < 0.001). More moderate but still significant correlations were observed for joint effusion, ESR, and platelet count. By contrast, IL-17, IL-1β, age, and synovial hypertrophy were not significantly associated with PMR-AS. Disease duration in weeks showed a weak inverse correlation with activity (rho = −0.219, *p* = 0.026), suggesting that higher inflammatory activity may be more prominent earlier in the disease course within this dataset.

Taken together, the correlation analysis indicates that disease activity in PMR is primarily aligned with a dominant inflammatory axis driven by SAA, CRP, IL-6, and other acute-phase and imaging variables. These findings support the existence of a coordinated inflammatory axis linking acute-phase reactants, selected cytokines, and ultrasound activity.

### 3.3. AI-Based Prediction of High PMR Activity

**Figure 2:**
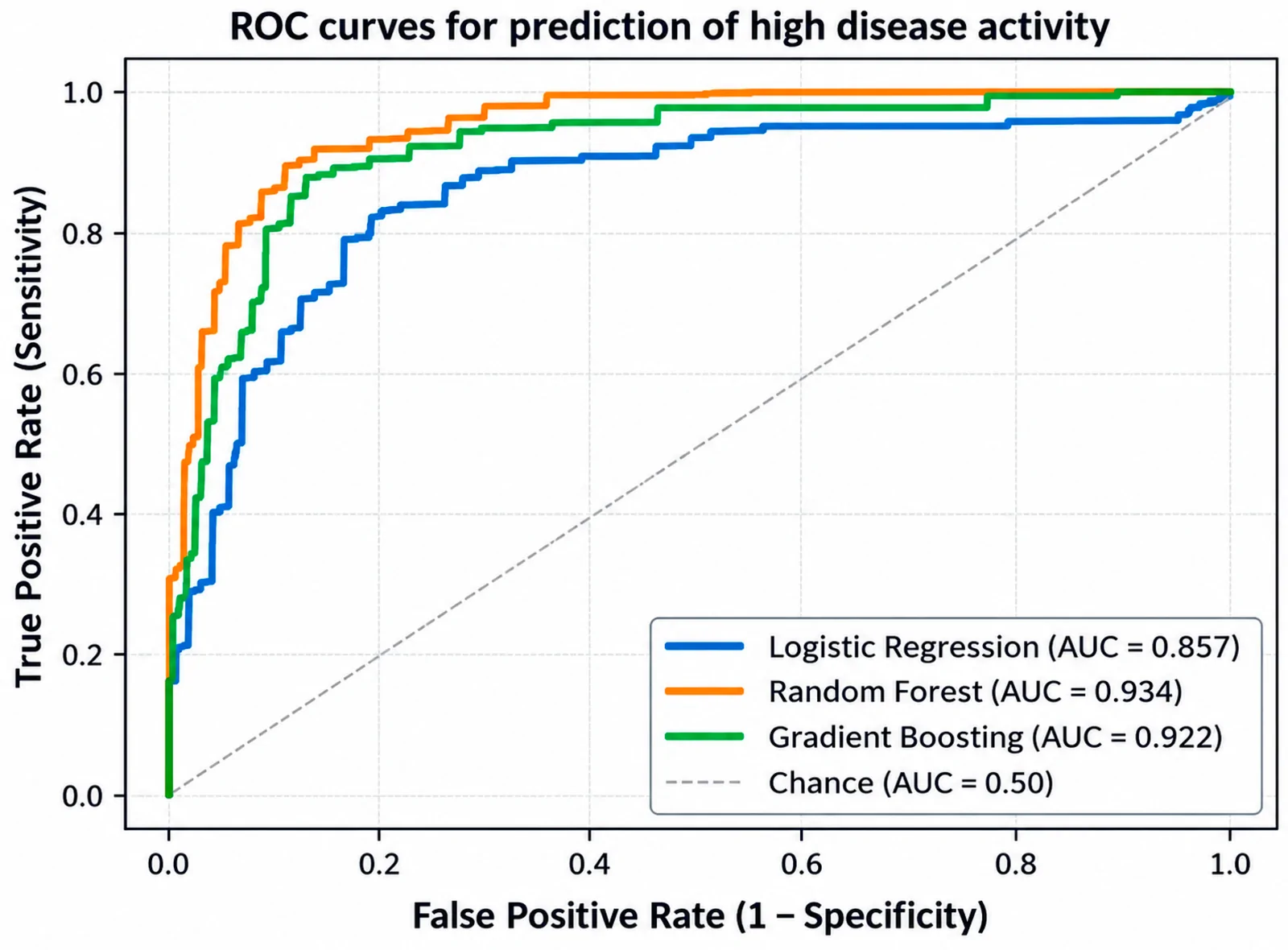
Receiver operating characteristic (ROC) curves for predicting high disease activity (PMR-AS ≥ 11). Random Forest demonstrated the highest cross-validated discriminative performance (AUC = 0.934), followed by Gradient Boosting (AUC = 0.922) and Logistic Regression (AUC = 0.857).

Supervised machine learning models were applied to evaluate whether the integrated clinical, laboratory, cytokine, and ultrasound dataset could discriminate patients with PMR-AS ≥ 11 from those with PMR-AS < 11. Model performance was assessed using stratified 5-fold cross-validation, and results are summarized in Figure 2 and Table 2. All three classifiers demonstrated good discriminative ability. Random forest achieved the highest cross-validated AUC (0.934), while gradient boosting showed a comparable performance (AUC 0.922) and yielded the highest overall accuracy (0.883). Logistic regression provided a slightly lower but still robust performance (AUC 0.857), indicating that a substantial portion of the predictive signal was already captured by a linear model, with further improvement obtained through non-linear ensemble approaches. While random forest achieved the highest discriminative performance in terms of AUC, gradient boosting showed the best overall balance between sensitivity and specificity, resulting in the highest accuracy and F1-score. In contrast, random forest demonstrated higher sensitivity, suggesting a greater ability to identify patients with higher disease activity, whereas gradient boosting provided slightly better specificity.

Overall, these findings indicate that disease activity in PMR can be reliably captured when clinical variables, inflammatory biomarkers, cytokine measurements, and ultrasound features are analyzed jointly within a multidimensional framework. Feature-importance analysis derived from the random forest model SAA as the most informative variable, followed by CRP, IL-6, fibrinogen, MRP, and Power Doppler signal. This ranking is consistent with the results of univariate and correlation analyses and suggests that high disease activity is best represented by a coordinated inflammatory profile rather than by a single parameter. However, these findings should be interpreted as exploratory and internally validated. External validation in independent cohorts will be necessary to confirm the generalizability of the observed patterns.

Together, these results support the concept of PMR activity as a multidimensional inflammatory construct that becomes more accurately captured when heterogeneous biological domains are analyzed in combination.

### 3.4. Sensitivity Analysis Using Continuous PMR-AS

To evaluate whether the principal findings depended on the exploratory dichotomization of PMR-AS, an additional sensitivity analysis was performed treating PMR-AS as a continuous outcome. A random forest regression model demonstrated good predictive performance (cross-validated R^2^ = 0.73; mean absolute error = 1.64 PMR-AS units). Importantly, the ranking of predictor importance remained largely unchanged compared with the primary classification analysis. SAA remained the most informative variable, followed by IL-6, CRP, fibrinogen, ESR, platelet count, and MRP. These findings indicate that the multidimensional inflammatory signature identified in the primary analysis was robust and was not primarily driven by the selected binary threshold.

### 3.5. Multivariate Structure and Unsupervised Phenotypes

**Figure 3:**
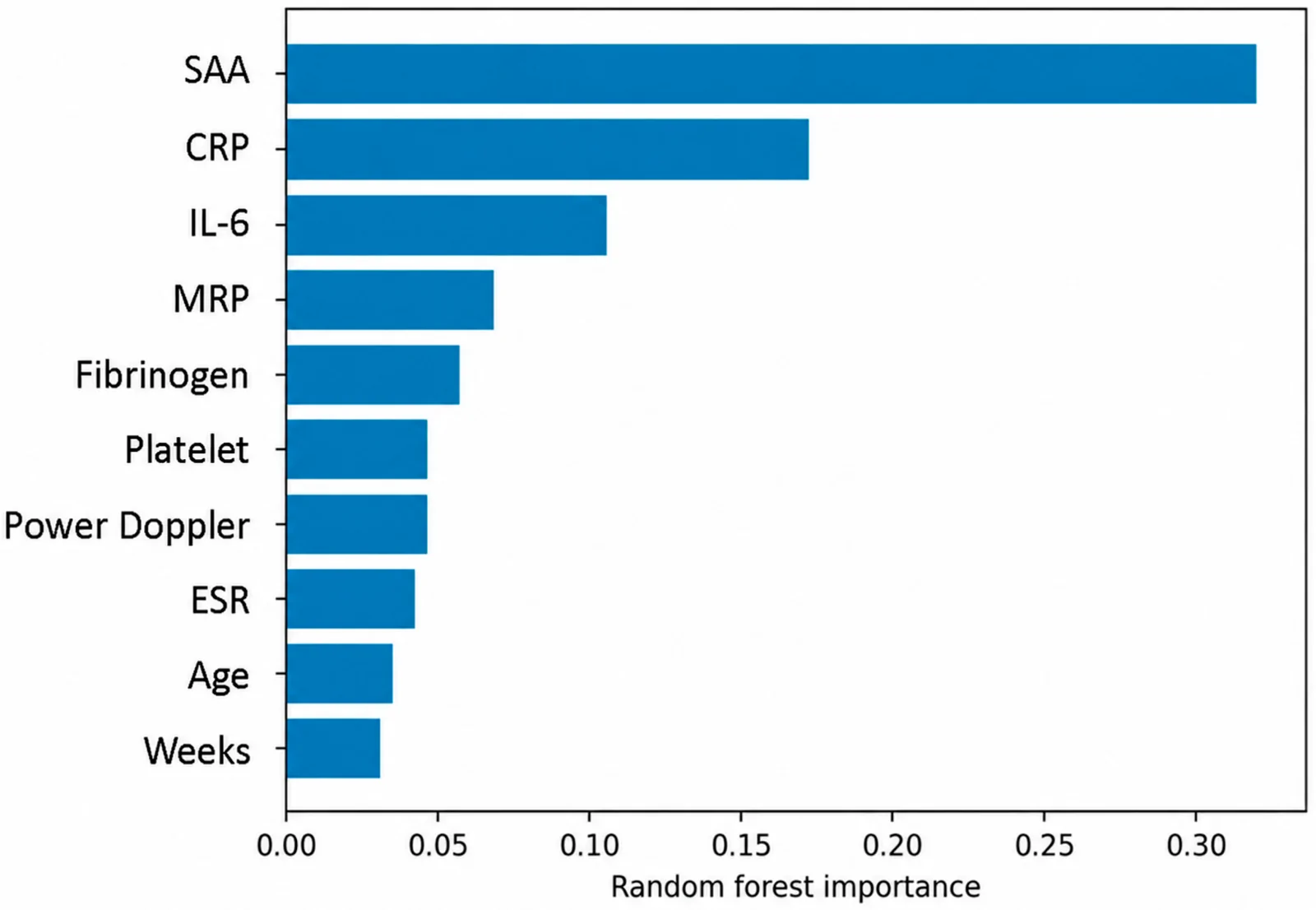
Random-forest feature importance plot. Within this dataset, SAA consistently showed the highest predictive contribution to high PMR activity across the evaluated machine-learning models, followed by CRP, IL-6, fibrinogen, MRP, and Power Doppler signal. However, feature importance reflects the relative predictive value of each variable within the model and should not be interpreted as evidence of biological hierarchy or causal relevance. Instead, the overall pattern indicates that PMR disease activity is best represented by an integrated inflammatory signature, in which acute-phase reactants, cytokines, and ultrasound findings provide complementary information rather than any single biomarker alone.

To better understand the relative contribution of individual variables to model performance, feature importance was assessed using the random forest classifier. As shown in Figure 3, SAA emerged as the most influential predictor, with a clear margin over all other variables.

Following SAA, the most informative features included IL-6, CRP, fibrinogen, platelet count, and MRP, with additional contributions from Power Doppler signal and ESR. This ranking is consistent with the results of univariate comparisons and correlation analysis, reinforcing the central role of systemic inflammation in defining disease activity. Notably, the inclusion of Power Doppler among the most relevant variables indicates that ultrasound-derived features provide complementary information beyond circulating biomarkers alone. In contrast, demographic variables and cytokines such as IL-17 and IL-1β contributed minimally to model discrimination.

### 3.6. Dimensionality Reduction and Exploratory Phenotypic Structure

**Figure 4:**
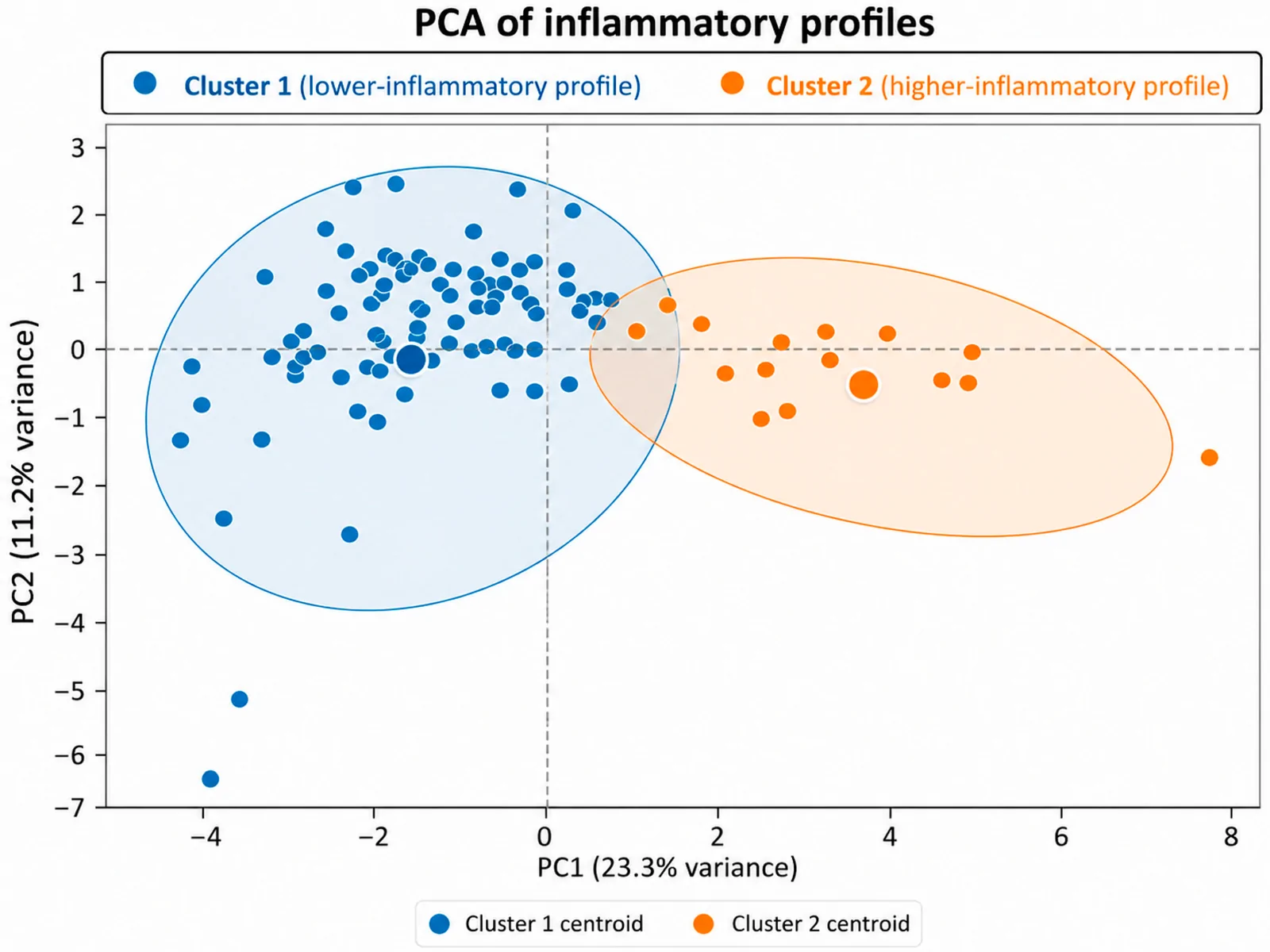
Principal component analysis (PCA) of the standardized dataset showing the separation of the two unsupervised clusters. The smaller cluster identifies a high-inflammatory phenotype associated with substantially higher PMR-AS values, whereas the larger cluster corresponds to patients with a lower inflammatory burden.

To explore the global structure of the dataset, PCA was performed after feature standardization. As shown in Figure 4, the first two principal components accounted for a meaningful proportion of the overall variance, with PC1 explaining 23.3% and PC2 explaining 11.2% of the variance. Inspection of the component loadings indicated that PC1 was primarily driven by inflammatory variables, particularly CRP, fibrinogen, SAA, MRP, ESR, and platelet count, with additional contributions from Power Doppler and joint effusion. This pattern supports the interpretation of PC1 as a global inflammatory severity axis. By contrast, PC2 appeared to be more influenced by ultrasound-related and structural variables, particularly joint effusion, Power Doppler, and synovial hypertrophy, suggesting a partially distinct imaging-related dimension.

In exploratory unsupervised analyses, clustering also suggested the presence of biologically distinct patient subsets. In particular, a smaller subgroup characterized by a more inflammatory profile showed markedly higher disease activity, with a mean PMR-AS of 15.9, compared with 9.3 in the larger subgroup. Although exploratory, this finding is consistent with the hypothesis that PMR does not represent a fully homogeneous inflammatory state, but rather a spectrum of activity patterns with potentially different biological underpinnings.

Taken together, these analyses indicate that disease activity in PMR is best captured by a coordinated inflammatory signature integrating acute-phase reactants, cytokine-related signals, and ultrasound features. Within this dataset, SAA consistently provided the greatest predictive contribution across the evaluated models. Nevertheless, feature importance should be interpreted as a measure of predictive relevance within the model rather than evidence of biological hierarchy or causality. The overall findings instead support a multidimensional representation of PMR disease activity, in which SAA, CRP, IL-6, and complementary laboratory and imaging variables jointly characterize the systemic inflammatory phenotype. This integrated pattern provides a more biologically coherent representation of PMR activity than isolated laboratory or imaging parameters considered independently.

### 3.7. Integrated Interpretation of Results

When considered together, the univariate comparisons, correlation structure, machine learning models, and dimensionality reduction analyses converge toward a consistent interpretation. Patients with higher PMR-AS are characterized by a multidimensional inflammatory profile dominated by marked elevations in SAA, CRP, and IL-6, accompanied by higher fibrinogen, platelet count, and more pronounced ultrasound inflammatory activity.

Across all analytical approaches, SAA was the most informative marker in relation to disease activity. At the same time, the combined performance of acute-phase reactants, cytokines, and imaging variables indicates that disease severity is better represented as an integrated biological continuum than as the isolated expression of a single laboratory abnormality.

## 4. Discussion

This study explores whether integrating clinical variables, inflammatory markers, cytokines, and ultrasound findings can provide a more informative representation of disease activity in PMR. Rather than relying on a single parameter, our results suggest that the relevant signal emerges from the combination of different inflammatory domains, including acute-phase reactants, IL-6-related pathways, and imaging evidence of active musculoskeletal inflammation. In this setting, machine learning should be interpreted as a tool to handle complexity, rather than as a replacement for clinical reasoning [18,19].

These findings must also be interpreted with due consideration of the limitations inherent in the current instruments for evaluating disease activity. Although the PMR-AS is among the most widely adopted and validated metrics, it exhibits strong yet imperfect discriminatory capacity, characterized by variable optimal cut-off values that are influenced by factors such as disease stage and concurrent treatments. Despite these complexities, the PMR-AS remains a valuable tool for monitoring disease activity and guiding clinical decisions, particularly when changes in its value between visits are considered [20].

Furthermore, the lower performance of individual components compared with the composite score highlights that no single parameter—whether clinical or laboratory-based—is sufficient to adequately represent disease activity, reinforcing the concept that PMR is an intrinsically multidimensional condition [8].

Among the variables analyzed, SAA consistently showed the strongest association with disease activity across different analytical approaches. Together with CRP and IL-6, SAA identified a coordinated circulating acute-phase inflammatory signature associated with higher PMR activity. This observation is biologically consistent with the well-recognized IL-6-driven acute-phase response in PMR [6]. At the same time, the present findings should not be overinterpreted. We do not suggest that SAA is universally superior to traditional inflammatory markers. Rather, within this cohort, it provided the greatest discriminative information among the evaluated biomarkers. Importantly, these circulating biomarkers should be interpreted as indicators of the systemic inflammatory response associated with disease activity rather than as comprehensive surrogates of PMR immunobiology, which also encompasses tissue-resident immune cells, stromal activation, and compartmentalized inflammatory processes that cannot be fully captured by peripheral blood biomarkers alone. The role of IL-6 observed in our analysis is also consistent with current knowledge of PMR pathophysiology. Its association with disease activity and its relevance as a therapeutic target have been well documented [3,4,5]. In our data, IL-6 remained among the most informative variables, although its contribution appeared to depend on its interaction with other inflammatory and imaging features. This reinforces the concept that PMR activity is better understood as a composite inflammatory state rather than the expression of a single pathway. In contrast, circulating IL-1β and IL-17 showed minimal discriminatory value in the present cohort despite being measured using highly sensitive ELISAs (lower limit of detection: 0.1 pg/mL for both cytokines). This observation should not be interpreted as evidence against the involvement of Th1/Th17 pathways in PMR pathogenesis. Rather, it is consistent with the concept of compartmentalized immune activation, whereby tissue-level immune responses may not be adequately reflected by circulating cytokine concentrations. Consequently, peripheral blood levels of IL-1β and IL-17 may underestimate the contribution of Th1/Th17-mediated immune mechanisms to PMR immunopathology. Indeed, accumulating experimental evidence indicates that PMR immunopathology extends beyond the circulating acute-phase response and involves tissue-resident macrophages, stromal cells, adaptive immune populations, and compartmentalized inflammatory processes within synovial and periarticular tissues. Consequently, circulating biomarkers provide an accessible measure of systemic inflammatory activity but cannot fully capture the biological complexity of the disease.

This aligns with recent clinical evidence, which highlights a significant unmet need in the management of PMR, with high relapse rates and difficulties in tapering glucocorticoid therapy. In parallel, the exploration of multiple therapeutic targets—beyond the IL-6 axis—further supports the idea that the disease is sustained by complex inflammatory networks not attributable to a single pathway [21]. Moreover, the observation of marked TH1 and TH17 cell infiltration at sites of disease suggests that circulating biomarkers may underestimate the intensity of local inflammation, reinforcing the rationale for integrating imaging and immunological data into models of disease activity. These findings further support the concept that circulating biomarkers reflect only one dimension of PMR pathobiology. Although acute-phase proteins and circulating cytokines provide valuable information on systemic inflammatory activity, tissue-level immune activation—including TH1- and TH17-mediated responses, stromal cell activation, and compartmentalized inflammatory processes—cannot be fully captured by peripheral blood biomarkers alone. These observations are particularly relevant in light of evidence showing that tissue inflammation in PMR is sustained by effector cells actively recruited to sites of disease through homing mechanisms involving receptors such as CCR6 expressed on TH17 cells and their CD161+ precursors [22].

Ultrasound findings provided additional, clinically meaningful information. Previous studies have shown that shoulder and periarticular ultrasound can improve the assessment of PMR [14,15,16], and our results support this view. In particular, Power Doppler signal contributed to identifying patients with higher disease activity, suggesting that imaging captures aspects of inflammation that are not fully reflected by circulating biomarkers [13]. In this context, recent ultrasonographic evidence underscores the prominence of extrasynovial inflammation in PMR. Suzuki et al. demonstrated that hyperemia detected by Power Doppler predominantly localizes to extrasynovial soft tissues adjacent to the subscapularis tendon—more extensively than articular synovitis—in contrast to elderly-onset rheumatoid arthritis with polymyalgic presentation [17]. Although these findings require further validation, they suggest that the spatial distribution of ultrasound signals provides insights beyond the simple detection of inflammation. Thus, imaging contributes not only to identifying inflammatory activity but also to characterizing its anatomical topography [17].

An important aspect of this study is the consistency of the findings across different analytical approaches. Group comparisons, correlation analyses, machine learning models, and unsupervised methods all pointed in the same direction, identifying a subgroup of patients with a more pronounced inflammatory profile and higher PMR-AS values. While this convergence does not eliminate potential biases, it strengthens the interpretation that the observed pattern reflects a real biological signal rather than a modeling artifact. Some limitations should be acknowledged. The study is based on a relatively small, single-center cohort, and the results may therefore be influenced by sample-specific characteristics. In addition, detailed information on comorbidities was not systematically available and was therefore not included in the present analysis. This should be considered when interpreting the findings, as comorbid conditions may influence systemic inflammatory biomarkers. The dichotomization of PMR-AS at the cohort median was a pragmatic methodological choice adopted to obtain balanced outcome classes for the exploratory machine-learning analyses and to minimize class imbalance. However, this threshold is data-driven and should not be interpreted as a clinically validated definition of high disease activity. To address this limitation, we additionally performed a sensitivity analysis using PMR-AS as a continuous outcome, which yielded a comparable ranking of the principal inflammatory variables, supporting the robustness of the identified multidimensional inflammatory signature independently of the selected classification threshold [1,23]. Furthermore, the absence of a standardized imaging protocol across all participating sites could introduce variability in ultrasound assessments, potentially affecting the generalizability of the findings regarding specific anatomical involvement. Despite these limitations, which mirror challenges in other PMR investigations such as small cohorts, single-center designs, retrospective biases, and ultrasound protocol variability [24,25,26], the alignment of imaging with clinical measures and the reproducible detection of inflammatory subgroups via diverse analytics indicate reliable contributions to understanding PMR heterogeneity. Therefore, further multicentric studies with larger sample sizes and standardized imaging protocols are warranted to validate these observations and to establish robust imaging biomarkers for disease stratification and therapeutic monitoring [25,27]. Beyond validation, future research should investigate the integration of advanced imaging modalities, such as MRI, to provide a more comprehensive characterization of tissue-level inflammation and to potentially differentiate various inflammatory arthropathies [28,29]. In addition, in our study the models were internally validated but not tested in independent cohorts, and the findings should therefore be considered preliminary. Future studies should focus on external validation and on longitudinal data to determine whether the identified markers can support monitoring over time or prediction of disease course. In particular, it would be relevant to assess whether integrating biomarkers such as SAA and IL-6 with ultrasound findings can improve clinical decision-making in routine practice. Overall, these findings support the view that PMR disease activity is associated with a coordinated circulating inflammatory network rather than the isolated behavior of single biomarkers. Although this integrated signature captures the dominant systemic inflammatory response, it should not be interpreted as a comprehensive representation of PMR immunobiology, which also involves tissue-level immune mechanisms beyond those measurable in peripheral blood.

## 5. Conclusions

In this exploratory single-cohort study, the integrated analysis of clinical, laboratory, cytokine, and ultrasound variables identified a coherent circulating inflammatory profile associated with higher PMR activity. Rather than reflecting the behavior of a single biomarker, this profile emerged from the combined contribution of acute-phase reactants, IL-6-related pathways, and imaging evidence of ongoing inflammation. Within this multidimensional framework, SAA provided the highest predictive contribution among the evaluated circulating biomarkers, although this finding should be interpreted as reflecting predictive performance within the present analytical framework rather than biological superiority.

Overall, these findings support the view that PMR activity may be more effectively characterized through coordinated inflammatory profiling than through isolated biomarkers considered individually. Importantly, the identified circulating inflammatory signature should not be regarded as a comprehensive representation of PMR immunobiology, which also involves tissue-resident immune cells, stromal activation, and compartmentalized inflammatory processes that are not directly captured by peripheral blood biomarkers. From a clinical and analytical perspective, integrating heterogeneous clinical, laboratory, cytokine, and imaging data may provide a more informative representation of disease burden than conventional approaches based on individual parameters.

Nevertheless, the exploratory nature of the present study should be carefully acknowledged. The analysis was conducted in a single cohort and lacks external validation; therefore, the findings should be interpreted cautiously and considered primarily hypothesis-generating. Future investigations should focus not only on independent validation but also on longitudinal assessment to determine whether multimodal inflammatory signatures can support dynamic disease monitoring and contribute to more personalized therapeutic strategies in PMR.

## Figures and Tables

**Table 1:** Baseline characteristics stratified by PMR activity.

Variable	PMR-AS < 11	PMR-AS ≥ 11	*p*-Value
Female sex, n (%)	29 (63.0)	36 (63.2)	1.000
Age, years	77.00 [71.00–81.00]	76.00 [71.00–80.00]	0.845
Disease duration, weeks	7.50 [6.00–12.00]	6.00 [6.00–8.00]	0.003
ESR, mm/h	21.50 [12.25–25.75]	28.00 [18.00–55.00]	0.017
CRP, mg/dL	0.35 [0.18–0.76]	1.59 [0.94–3.48]	<0.001
Fibrinogen, mg/dL	443.00 [389.25–510.00]	512.00 [450.00–630.00]	0.001
Platelets, ×10^3^/µL	243.50 [209.25–279.25]	269.00 [228.00–311.00]	0.012
IL-6, pg/mL	3.75 [3.40–6.28]	11.30 [6.60–21.80]	<0.001
SAA, mg/L	10.00 [6.90–17.35]	92.00 [45.50–192.00]	<0.001
MRP, µg/mL	1.70 [1.32–1.95]	2.30 [1.70–3.50]	<0.001
IL-17, pg/mL	0.10 [0.10–0.10]	0.10 [0.10–0.10]	0.875
IL-1b, pg/mL	0.10 [0.10–0.47]	0.10 [0.10–0.40]	0.958
Joint Effusion, score	2.00 [1.00–2.00]	2.00 [2.00–2.00]	<0.001
Synovial Hypertrophy, score	0.00 [0.00–0.75]	0.00 [0.00–1.00]	0.466
Power-Doppler, score	2.00 [1.00–2.00]	2.00 [2.00–2.00]	<0.001

Values are reported as median [IQR] unless otherwise specified. Sex is reported as female n (%).

**Table 2:** Cross-validated performance of machine learning models for prediction of PMR-AS ≥ 11.

Model	AUC	Accuracy	Sensitivity	Specificity	F1-Score
Logistic Regression	0.857	0.816	0.807	0.826	0.829
Random Forest	0.934	0.864	0.895	0.826	0.879
Gradient Boosting	0.922	0.883	0.877	0.891	0.893

## Data Availability

The data supporting the findings of this study are available from the corresponding author upon reasonable request.
